# Editorial Chit-Chat

**Published:** 1877-04

**Authors:** 


### Editorial Chit-Chat.

ÆSTHETIC.—A lady that we wot of, has her potatoes scrubbed with a tooth brush—a *new* one, so she declares.

THE NEW COVER.—We had thought of presenting a handsomly engraved cover for our journal with this number, but have as yet been unable to procure a design that suited us. We hope to have it completed for our next volume.

FLOWER TIME.—Soon time to make garden. If you have not space for flowers and vegetable garden both, have the former to the exclusion of the latter. Vegetables can be much cheeper purchased than cultivated, but flowers not only afford you excellent out-door employment, but reward you a hundred fold, by their beauty and fragrance.

"GEMS OF THE DANCE."—Such is the title of a book of instrumental music, published by Oliver Ditson & Co., of Boston. It is a book of brilliant music, containing waltzes, golops, polkas, quadrilles, &c., by Strauss, Zikoff, Faust and other celebrated composers. The book has 232 pages of music and sells for $2.50. Cheap and desirable.

H.—We regret our inability to furnish our readers with any more of the witty sayings of our friend H. He is still about, has lost not of his talent for story telling and sparkling sayings, but is as dumb as a hatchet when he sees the editor at his table with pencil in hand. When we get him in camp, he will let fly as usual, when we will try catch some of the good things he says and dish them up for the July number.

ART PARLOR.—If one be of art arty, and of heart hearty, let him betake himself to the exquisitely arranged, munificently appointed, oriently furnished, elaborately garnished, generously divanned and altogether artistically adorned suite of apartments in which our cosmopolitan friend, Frank Hall, modestly invites his less traveled friends to loaf an hour away, and muse themselves into an ecstacy—a dream of the orient—where the gorgeous East with richest hand, showers on her kings barbaric pearl and gold. One can well imagine himself while there, amid the wealth of Ormus or of Ind, and come out therefrom as one comes out from a siesta to which he has been invited by the god Opos.

A LITTLE LATE.—By reason of the illness of the editor, this issue of the BISTOURY has been a little delayed. And while speaking of delays, let us remind our readers that before the July number appears, we take our yearly vacation, spending the month of June in the woods. Be not surprised, therefore, if the BISTOURY does not come to hand before the 15th of the month, for our vacation we *must* have.

TIME ABSORBENT.—If an editor or a doctor has twenty-three hours and a half to spare of the twenty-four, and has been conjuring up all sorts of methods and devices for filling up that little balance of time, we think we can help him out by a mere suggestion—get a conservatory and run it. The beauty of a conservatory is its capacity for absorbing unlimited time. Next to a houseful of low-down grates, or a family of ten children, there's no time absorbent like unto a conservatory.

BILE TIME.—The quinine days have come again, the achieist of the year, when every bone seems in a vice, and sing-sing goes your ear; when appetite has gone to roost, and every minute seems an agonizing torture, and sleep is full of dreams; when life's not worth the living for, and one feels like to die, yet fears he won't, then hopes he won't, and gets well by and by. Such are among the Spring's delights that come from year to year—alas! the quinine days *have* come the queerest of the queer.

HORSEBACK RIDING.—The practice of horseback riding has become quite a mania in our city. Early every morning, through mud and slush, may be seen a bevy of young and middle aged people taking their morning exercise. Nothing like it. If every man who owns a horse would ride instead of drive him, it would be the means of dispelling much of the indigestion and dyspepsia with which the nation is afflicted. If you would be free from the ills consequent upon indigestion, eat moderately, arise early, and take a two hours horseback ride before going to business. No bottled medicine can approach such a prescription in efficacy.

THE SPRING STYLES.—The several establishments in this city where ladies hats are exhibited and sold, have had their openings, and, judging from the brilliant display of head-gear at church, these fine Sunday mornings, we should say the stock had been entirely disposed of. And *such* bonnets! As works of art, they are magnificent

—splendid in harmony of colors—gorgeous in ribbons, feathers, silk, velvet, and flowers. But as a protection for the head, worn as a covering, for the same reason that a man-wears a hat, they are sublimely rediculous. Take your seat well back in church, and see them pass. Hats with feathers—hats without; hats with ribbons—hats with none; hats with feathers, flowers and filigree—hats plain; hats on top the head—hats on the side—hats over the eyes—hats suspended from the ear—hats perched upon the extreme end of the knot of hair on back of the head, making you feel anxious for its safety; in truth, all kinds, shapes and degrees of hats. They are a sight to behold. Go to Richards & Chase's neat little bower of a store, and see them artistically arranged. It is a sight worth the visitation of many miles to see.

---

STRONG.—H. has waked up, could n't hold in any longer, and has delivered himself of the following story:—Years ago, it was customary to furnish liquor in the harvest fields. H's father was a temperance man, and refused to furnish his laborers the required stimulant, in consequence of which, he experienced considerable difficulty in securing the necessary help. One day a stalwart, raw-boned, Yankee appeared at the harvest field fence, and asked for a job. H's father informed him of his temperance proclivities and of his determination not to furnish any liquor. Applicant offered no demur, but signified his inclination to work. The old gentleman then enquired whether he was well and strong, able to do a full day's work. "Strong! Strong!" exclaimed the laborer, "why, I have taken my father by the throat, downed him, and choked him black and blue in the face, many a time, and he's a third heavier than you." Applicant was rejected.

---

WEATHERY.—Don't know how it is with you folks off East, or down South, or out West, but, speaking of the weather, we have a decided opinion that right here, in this particular portion of the Empire State, we literally—not slangfully—know how it is ourselves. For a very spiteful spell of the spitefulest gustiness in the way of snow, wind, rain and other meteorologic phenomonality, we can claim, for the closing week of March, one thousand eight hundred and seventy-seven, the most honored palm in the whole range of the *chamoerops humilis*. If any habitable tract on the surface of this mundane projectile thinks it can show a better betting hand than this immediate latitute can, in this respect, we are authorized to say that we are not afraid to "see it" and go a little something better. It has started up all of last year's rheumatisms—rejuvenated all the midwinter coughs—set all the noses to running—increased wash-lists to the maximum in the handkerchief line—raised the price of overshoes and breast-pads—started new fuel bills, and stopped the influx of "Odes to Spring," that gentle ethereality of whom all poets become enamored at just about this season of the year. For this latter we are duly thankful—for this, if nothing more.

---

FOND OF IT?—How, sharper than a serpent's thanks it is to have a toothless child! or words to that effect, but Oh, oh, ouch! how sharper than a pin of burnished steel it is—to an editor in a hurry to get out his past due periodical—to have a terrapin. If thou hast an editor for thine enemy, make him a gift, and let it be a big fat terrapin; for behold he will go to, construct a soup thereof, invite his friends to join him about his mahogany, and all the little diabolic imps in the spirit land of the Bohemian will snap their abdominal cartilages with cachinnatory convulsions over his cranial tortures for a quartette of days and nights, while he who presides over the ink-pot cries in his ears, copy! COPY! COP-PEE! From these three things, good world, deliver us: the book-agent, the insurance solicitor and the terrapin man. Miseries are a plenty, but as to miseries, the chiefest of these is terrapin.

---

THE TELEPHONE.—No invention of the century is more wonderful, more ingenious than the electric apparatus by which we are enabled to talk by telegraph. An invention that enables one in New York to hold conversation with, and recognize the voice of a friend in Philadelphia, is more than wonderful. Recently a concert was given in Philadelphia and listened to and enjoyed in Steinway Hall, New York, by means of the telephone. Soon, when you have occasion to leave home and go across the continent, you can sit down in some cosy office and hold a conversation with wife or sweetheart, and scarcely discover that you have left home at all.

Should the telephonist be a handsome girl, young and pretty, you can place your arm about her waist, (if she'll let you), listen to the loving words of the girl at the other end of the line, and enjoy all the delights of home comfort. Wife could convey baby to the instrument that you might recognize its little cry, and determine from its vehemence whether it had colic or was only teething. The possibilities of the telephone are beyond compare,—not only can we have gas and heat supplied our dwellings from a common reservoir, but music will be turned into our houses in the same manner, so that at an evening party, you can have your music supplied from Paris or New York at so much a yard or measure. Wonderful and passing strange are the inventions of the age.

---

A MEDICAL BILL OF FARE.—There exists in this city, a choice club of congenial and happy fellows, who, once a week, congregate at the dwelling of one of their number to play the ancient, but exceedingly interesting game of "old sledge." During the evening, the host serves a luncheon, partaking of more or less excellence, depending upon the capabilities of the stomachs of the members. The last meeting occurred at a physician's house, when one of the party furnished a bill-of-fare which is grotesque in its ridiculousness. Here it is:

COMPLIMENTARY SUPPER TO THE OLD SLEDGE CLUB.

*Tuesday, March 20th*, 1877.

POTAGES.

Terrapin Soup—Sitting on a Log. Filets de Angina Pectoris.

HORS D'ŒUVRES.

Spinal Curvature, a la Richard III.

RELEVES.

Trichina Spiralis, aux Champignons.
Tapeworm, a la Berner.
Varicose Veins en belle vue.

LEGUMES.

Pumpkin Seeds, Sea Weed, Castor Oil Beans.

ROTIS.

Saddle of Hip Disease. Diseased Joints.

ENTRETREMENTS.

Pickled Club Feet.
Cross Eyes and Carious Teeth, *assortis.*
Polypi.

VINS.

Rheumatic Mixture, Ginger Tonic, Soothing Syrup, S. T. 1860, X.

On the reverse of this card, was printed the toasts, and the names of the parties who were expected to respond. We have not the space to give the array of subjects—they were queer, suggestive and comical.

As might be expected, some of the members were unable to report for duty for several days after the feast, and all laid it to the terrapin.

---

FRITZ' SAYINGS.—Fritz is a five year old, as lively as a cricket, and as mischevious as a monkey. Like most boys of this character, he has his own ideas of matters and things, and does not hesitate to express them upon any and all occasions. At the recent election, he heard much said about the polls, and was an earnest observer of politics, being at all the pole raisings in the neighborhood and invariably shouting for his favorite candidate. The Democratic pole was located on a corner, and a short distance away, in front of the usual place for voting, stood the Republican pole. On election day, Fritz rushed into the dining-room flushed with excitement, and exclaiming at the height of his voice—"Hayes' has got it, Hayes' has got it for sure—'cause there's ever so many more men around the Republican pole than there is around the Democrats' pole." "Hurrah, why don't you," he shouted in his brother's ear, as he gave him a poke with his fist in his stomach, just by way of emphasizing the matter.

Fritz has been charged not to play on the railroad. One of his playmates was sadly injured while so doing, loosing both his feet. This very greatly distressed Fritz, and his father sought to impress the danger upon his mind more strongly by calling him in to witness an amputation of an arm of a man injured upon the road. Says the father, "Fritz, here's another person injured by playing on the railroad." The boy looked him over a moment, and then expressed himself in this manner:—"Humph, he's old enough to know better," and then proceeded to interest himself in the amputation.

---

BLUE GLASS MANIA.—There is no accounting for the whims of people. They are gregarious, and like sheep, will follow almost any old fool of a bellweather, if only for the sake of becoming as rediculous as he. A chap who is extremely fond of being called "General," who possessed a deaf mule and a rheumatic one at that, caused a beam of blue light to fall upon the tips of that hybrid's ear, when, behold, he was made to hear and his rheumatism disappeared. Upon this testimony, and the recital of other cases equally trustworthy, thousands of people are bespangling their windows with blue glass, and the marvelous results are becoming apparent.

One reliable correspondent writes us that a blue light of glass accidently placed in his front door transom, caused a ray of blue ray to fall upon the left hind leg of a cast iron bull dog, which kept watch in the hall. That dog deliberately walked off and has not been seen since. A German in this city introduced it into the window of his wife's sitting room. Last week she presented him with triplets. That window now looks as though a hail storm had struck it, and the glaziers are mending it with white glass. A lady's canary bird, hanging in the blue light of her setting room, in one week, became a blue-jay. A conservatory, in which the posts and rafters are of pine and hemlock, have all bristled with the green needles peculiar to them, and have grown so tall as to lift the roof many feet in the air—all by reason of the blue glass in the sash. There is at present no telling where and when the furore may terminate. Ladies are wearing blue stockings, (so we are informed), the girls wear blue veils—the boys blue spectacles, neck-ties and noses—the babies blue sashes—while the fathers look blue at the result.

But soon the delusion will be dispelled, when we will be content to sit and luxurate in the cheerful sunlight as of old, and settle down ready to be gulled with the next rediculous hobby that presents itself.

---

CONSERVATORY LABORS.—When we constructed a conservatory and filled it with beautiful plants, thinking that it might afford us pleasant recreation—a sort of resting spell from our professional duties, we did not clearly understand that we had enlisted for the war,—a warfare against green fly, apis, red spider, mealy bug, scale snd several other infernal nuisances, the precise value of which, in the economy of nature, we have signally failed to fathom or appreciate. We have tobacco smoked that little heathen of a green fly, until our face is the color of a well smoked mearschaum, while our whiskers are so bedewed with the filthy stuff that, our daughters hold their noses while they kiss us, and sneeze immediately after the ceremony. We fumigated with sulphur, for that cussed red spider, until we wheeze like a wind-broken horse, and our eye lashes have dropped out from being sprayed with whale oil soap while in search of mealy bugs.

We scarcely know which diversion we enjoy most—chasing the red spider up fifteen feet of *convolvulus major*, with a lighted brimstone match, and see him—and by "him" we mean millions of 'em—run to the very top of his ladder, then stand on his head, and while he makes all sorts of grimaces, kicks at you with his several and interesting hind legs; or to pour lime water into a flower pot and witness the antics of the worms who suddenly appear from the depths below—bag and baggage—and wiggle and squirm into more salubrious quarters. Sometimes we think it is worth the while after all, to interview these numerous little wretches. When we feel particularly "blue," as though life was scarcely worth the saving,—that all is headache and vexation of spirit,—that the world is full of poor and wretched people, and no means at our command for relieving them, we just run out and pour a pint of lime water into a flower pot—light a smudge in our tobacco kettle, and while the worms squirm, look cross-eyed and twist their tails into grotesque knots, and the green fly folds his wings, looks heavenward and sighs, we revel in delight and are happy that they are miserable too—for misery loves company,—confound them.

---

THE TEMPERANCE MOVEMENT.—We are again visited with a revival in favor of temperance. This time, it comes in a more acceptable form than the furore of a couple of years ago. Then, it was a warfare against the liquor vendors, now, an appeal to the inebrates themselves.

Many persons of influence and prominence in our community are joining the abstinence party and signing their names to the pledge not to drink intoxicating liquors again. This is as it should be. We hope the excitement will spread until every one in the land—old and young, will sign the pledge, for total abstinence is the only hope of sobriety. If the drinking men who are freely entering the temperance ranks will only abide there—*will keep their pledges*, a grand work has been accomplished. But whether they do or not, the influence that has been exerted over them while they remained sober, cannot but leave a favorable and lasting impression.

We have little or no faith in the possibility of reforming a habitual drunkard. There have been a few of this class who *have* reformed and made excellent citizens, but as a rule, the experiment is a failure, for chronic alcoholism is considered and has been proved to be an incurable disease. But, our young people are within the possibility of saving from this terrible vice, and toward them should our exertions be directed. They must be induced not only to sign the pledge, but to keep it as well. And in this matter, the ladies can be of incomparable help. They can, if they will, render drinking so unpopular that no young man would be guilty of the act. Let them discountenance it in every possible manner, by refusing the company of all those who drink,—by leaving the room when wine or liquors are introduced,—by shunning a young man who is seen to emerge from a saloon, and in any other manner that they may choose to exhibit their intolerance of drink and drinking people. Let the old bummers die off and go to their reward; they are without hope or means of succor, but the young people can be saved, and we hail with pleasure any means that is likely to render them "total abstinence" disciples. Therefore God speed the temperance movement.

